# One-year observation of leflunomide effectiveness and saftey in juvenile idiopathic arthritis : a clinical practice review (preliminary data)

**DOI:** 10.1186/1546-0096-9-S1-P176

**Published:** 2011-09-14

**Authors:** V Torrente-Segarra, P Santín, L Casas, S Ricart, R Bou, JB Ros, N Rodríguez, J Antón López

**Affiliations:** 1 Pediatric Rheumatology Unit, Hospital Sant Joan de Déu, Barcelona, Spain; 2 Rheumatology Department, Hospital General Hospitalet, Barcelona, Spain; 3 Rheumatology Department, Hospital Nuestra Señora Candelaria, Canarias, Spain; 4 Rehabilitation Department, Hospital Sant Joan de Déu, Barcelona, Spain

## Objectives

To assess efectiveness and safety of leflunomide (LFN) in juvenile idiopathic arthritis patients (JIA).

## Methods

We performed a retrospective medical charts review in all JIA patients who received LFN between Jan 2006 to Mar 2011 from our outpatient Pediatric Rheumatology Clinics. We collected data of: gender, age, type of JIA, date of onset, date of diagnosis, date of first DMARD, uveitis, reason to indiciate LFN, concomitant therapies, reason to discontinuation, joint activity (tender and swollen, low rang of motion count), and biological markers at baseline, 3 and 12-months follow-up.

## Results

We found 32 patients had used LFN sometime. Patients received oral 20 mg/daily LFN as compassionate therapy after methotreate use (at conventional dosage of 10-15mg/m2), in all cases. We present data from the first 14 patient medical charts reviewed. Female 11 and 3 Male; 7/14 olygoarticular ANA positive, 3/14 uveitis; 11/14 maintained LFN after one year-follow up. Reason: 5 due to partial response, 5 due to adverse event, and 4 due to intolerance, to methotrexate. 7/14 received combined therapy with etanercept, and 3/14 with infliximab. 10/14 (71%) received LFN for at least one year, 2/14 (14%) withdrawn LFN use due to clinical remission.

**Table:** 

*median	baseline	3 M	12 M
SJC	2.54	0.64	0

TJC	2.61	1.07	0.07

LRMC	2.14	1.57	0.92

## Conclusions

71% of patients who received LFN showed efficacy and only 14% had adverse events (recovered). Most were ANA positive oligoarticular JIA, and 2 more had uveitis.

Safety seemed acceptable either alone or with antiTNF concomitant use.

